# The Effectiveness of Adequate Antenatal Care in Reducing Adverse Perinatal Outcomes: Evidence From a Low- or Middle-Income Country

**DOI:** 10.7759/cureus.51254

**Published:** 2023-12-28

**Authors:** Mst. Nazmunnahar Mina, Mostafa Nuruzzaman, Mostafa Nahian Habib, Mahin Rahman, Faiza Mehrab Chowdhury, Syeda Nafisa Ahsan, Fabliha Fyrose Ahmed, Shajeda Azizi, Nazirum Mubin, A. H. M. Golam Kibria, Ferdous Ara Shuchi

**Affiliations:** 1 Obstetrics and Gynaecology, Delta Medical College & Hospital, Dhaka, BGD; 2 Anaesthesia, Analgesia and Intensive Care Medicine, Bangabandhu Sheikh Mujib Medical University, Dhaka, BGD; 3 Medicine and Surgery, Assasuni Upazila Health Complex, Satkhira, BGD; 4 Epidemiology and Biostatistics, Centre for Medical Research & Development (CMRD), Dhaka, BGD; 5 Radiotherapy, Dhaka Medical College Hospital, Dhaka, BGD

**Keywords:** adequate antenatal care, adverse perinatal outcomes, preterm birth, perinatal death, cesarean section

## Abstract

Background and aim

Antenatal care (ANC) is universally acknowledged as an essential intervention for enhancing the well-being of both mothers and children. The World Health Organization advises a minimum of four ANC visits. The objective of this study is to assess the effectiveness of adequate ANC in mitigating adverse perinatal outcomes.

Methods

This cross-sectional study was done at the Department of Obstetrics and Gynecology, Delta Medical College & Hospital, Bangladesh, from March 2023 to August 2023. A total of 226 mothers who gave birth at the hospital during this period were enrolled in the study.

Results

More than 87% of the participants received adequate (≥4 visits) antenatal care from a registered physician. More than 84% of the mothers gave birth via cesarean section. Among the mothers who received inadequate ANC, the proportion of adverse perinatal outcomes was higher (69.0%) than that of those who received adequate ANC (32.0%). A significant association (p<0.05) was noted between inadequate antenatal care and adverse perinatal outcomes. Pregnant women receiving adequate antenatal checkups were 79% less likely to experience adverse perinatal outcomes compared to those receiving inadequate ANC.

Conclusion

Adequate ANC is a very efficient and economical strategy for mitigating adverse perinatal outcomes.

## Introduction

Conceiving a child and giving birth still carry substantial dangers for both mothers and children, especially in low- and middle-income countries (LMICs), despite widespread efforts to improve global accessibility to basic healthcare for women. The decline of maternal as well as newborn child mortality rates continues to be a priority on the agenda for global development, as evident in its inclusion in the United Nations' Sustainable Development Goal (SDG) 3 [1].

Annually, close to three million infants die within the first month of life, and a substantial proportion of these fatalities and medical conditions in LMICs result from preventable causes [1,2]. In Bangladesh, the documented neonatal death rate is 24/1000 live births and the stillbirth rate is 36/1000 live births [3,4]. It is worth mentioning that a significant proportion of these deaths could have been prevented [5]. The rising trend in perinatal mortality is related to elevated stillbirth rates and fewer antenatal visits, according to studies [6]. Antenatal care (ANC) is globally recognized as a crucial intervention for maternal and child health [7]. The World Health Organization (WHO) suggests a minimal number of four ANC visits (adequate ANC), commencing in the first trimester of pregnancy, as a recommendation to mitigate maternal and neonatal morbidity and death [8]. ANC has a great role in reducing the risk of stillbirth, reducing neonatal mortality, enhancing the nutritional requirements of children, and minimizing newborn admissions to the neonatal intensive care unit (NICU)[1,9,10].

Treating a preterm baby or neonate who needs NICU support is costly in Bangladesh, with a minimum cost of $50-60 per day [11]. On top of that, with no government health insurance policy, families need to carry the entire cost of treatment. On the other hand, the average unit cost of ANC visit provision ranges from $0.78 to $2.75 in rural areas in Bangladesh [12], which might be a bit higher in urban areas. But a point to be noted here is that more than 50% of mothers in Bangladesh still do not receive adequate ANC [13]. This imparts considerable adverse outcomes. This study aims to explore adverse perinatal outcomes and examine the effectiveness of ANC on perinatal outcomes from a Bangladeshi viewpoint to generate evidence and educate pregnant women who are not receiving appropriate ANC.

## Materials and methods

This cross-sectional study was conducted at the Department of Obstetrics and Gynecology, Delta Medical College & Hospital, Dhaka, Bangladesh, from March 2023 to August 2023. Ethical clearance was granted by the Institutional Ethics Committee of Delta Medical College & Hospital (Approval number: DLMC/IEC/202/3). Informed written consent was obtained from all participating mothers after a comprehensive explanation of the study’s objectives and procedures. All mothers who gave birth at that hospital during the study period were considered for inclusion in the study. Mothers without ANC records were excluded. Considering the proportion of mothers receiving regular ANC of 21% reported by El Arifeen et al. and the Z distribution at a 5% level of significance and an allowable error of 6%, the calculated sample size was estimated to be 178 ​​​​[14]. However, there was an opportunity to get an increased number of pregnant women within the study period. A total of 226 mothers meeting the inclusion criteria willingly participated in the study. The participants were recruited utilizing a purposive sampling technique. A pre-tested questionnaire was used to gather data through in-person interviews, looking into ANC records and delivery notes.

Obstetric history, including gravidity and parity, along with gestational age at the first ANC visit, the total number of ANC visits, laboratory investigations, weight (kg), and blood pressure on each ANC visit were collected from the ANC record and documented. Adequacy was defined as a minimum of four ANC visits commencing in the first trimester of pregnancy. Information regarding the perinatal outcomes, encompassing preterm birth, less fetal movement, fetal distress, low birth weight, intrauterine growth retardation (IUGR), admission to the neonatal care unit, and perinatal death, was obtained from the delivery notes. A newborn with a weight <2500 grams at birth was considered to have a low birth weight and preterm birth as delivery occurred at <37 weeks of gestation [15]. IUGR (estimated fetal weight (EFW) <10th percentile), less fetal movement or fetal distress, were taken into account if clinical and ultrasonographic evidence were available [15]. Perinatal mortality was considered if death occurred from 28 weeks of gestation up to seven days of birth [16].

Statistical analysis 

The data was entered and cleaned using Microsoft Excel (Microsoft Corporation, Redmond, Washington, United States). The statistical analyses were conducted using IBM SPSS Statistics for Windows, Version 25.0 (Released 2017; IBM Corp., Armonk, New York, United States). Continuous variables were summarized using mean and standard deviation or median (range). Categorical variables were summarized using frequency and percentage. Chi-square test was done to determine the association between ANC and perinatal outcomes. Logistic regression was employed to evaluate the correlation between the adequacy of ANC and adverse perinatal outcomes. P-values <0.05 were considered statistically significant.

## Results

The mean age of the participants was 27.98 ± 5.01 years. Three-fourths of them were housewives. More than one-third were graduates (Table 1).

**Table 1:** Sociodemographic characteristics of the study participants (N=226) Data presented as frequency, percentage, mean ±SD, median (IQR) IQR: interquartile range

Demographic Characteristics		Frequency	Percentage
Age (years)	≤20	15	6.6
	21-30	138	61.1
	>30	73	32.3
	Mean ±SD	27.98±5.01	
	Median (IQR)	28 (24-32)	
Residence	Rural	49	21.7
	Urban	177	78.3
Religion	Islam	214	94.7
	Hinduism	12	5.3
Education	Up to primary	15	6.6
	Secondary	32	14.2
	Higher Secondary	77	34.1
	Graduation	86	38.1
	Post Graduation	16	7.1
Occupation	Housewife	170	75.2
	Service	24	10.6
	Student	22	9.7
	Others	10	4.4

**Figure 1:**
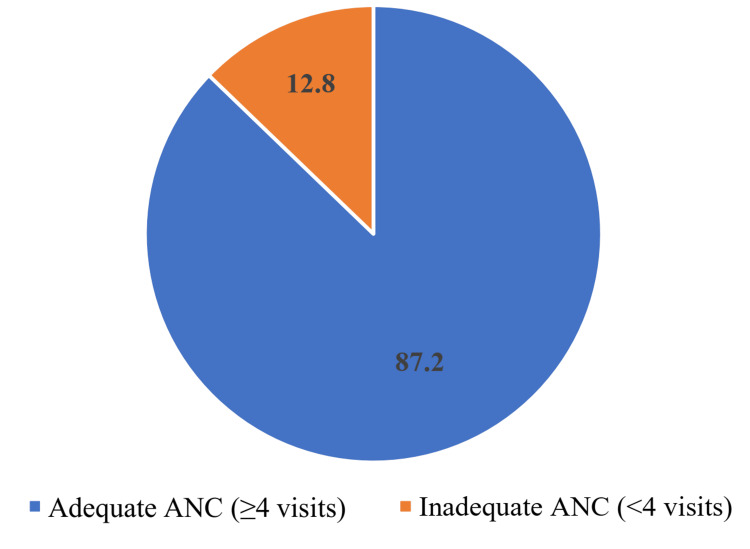
Proportion of participants receiving adequate/inadequate antenatal care

More than 87% of participants adequately received ANC (≥4 visits) from a registered physician (Figure 1).

One-third of the participants were primigravida. And more than 84% of the participants gave birth to the baby by cesarean section (Table 2).

**Table 2:** Obstetrical history of the participants (N=226) Data presented as frequency, percentage

Obstetrical history		Frequency	Percentage
Gravidity	Primigravida	76	33.6
	Multigravida (2-4)	138	61.1
	Grand multipara (≥5)	12	5.3
Parity	Nulliparous	93	41.2
	1-3	130	57.5
	≥4	3	1.3
Delivery	Vaginal	35	15.5
	Cesarean section	191	84.5

**Figure 2:**
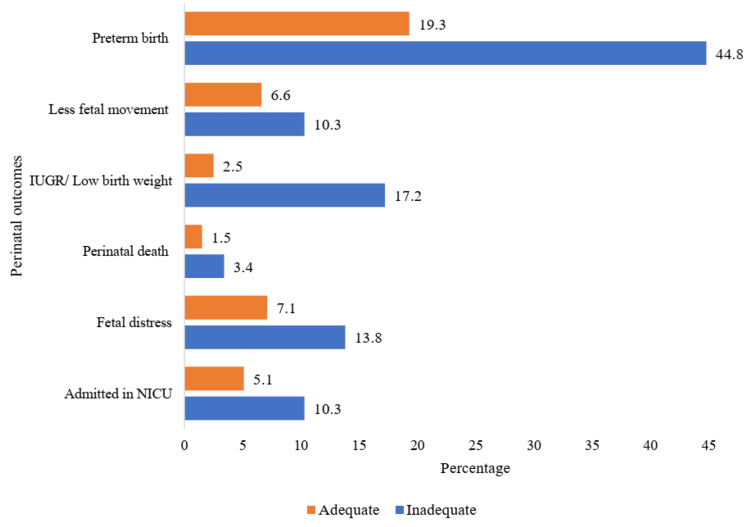
Distribution of adverse perinatal outcomes NICU: neonatal intensive care unit; IUGR: intrauterine growth retardation

Mothers who did not receive adequate ANC suffered from a higher proportion of adverse perinatal outcomes than those who received adequate ANC (Figure 2).

A significant association (p<0.05) was noted between inadequate ANC and adverse perinatal outcomes (Table 3).

**Table 3:** Association of antenatal care and perinatal outcome (N=226) A Chi-square test was done

Antenatal care	Perinatal outcome		Total	p-value
	Adverse	Normal		
Adequate (≥4 visits)	63 (32.0)	134 (68.0)	197 (100.0)	<0.001
Inadequate (<4 visits)	20 (69.0)	9 (31.0)	29 (100.0)	
Total	83 (36.7)	143 (63.3)	226 (100.0)	

There was no notable association found between demographic characteristics (such as maternal age, education, occupation, residence, and religion), maternal comorbidities/complications (including gestational diabetes mellitus, hypertension, hypothyroidism, anemia in pregnancy, and oligohydramnios), and perinatal outcome (p-value: >0.05) (Data not shown). Logistic regression signifies that adequate ANC acts as a protective factor against adverse perinatal outcomes. Compared to pregnant women who had inadequate ANC, those who received adequate ANC were 79% less likely to have adverse perinatal outcomes (Table 4).

**Table 4:** Logistic regression for the development of adverse perinatal outcomes in adequate antenatal care Data presented as odds ratio, 95% confidence interval

Factor	Odds ratio	95% Confidence interval		p-value
		Lower	Upper	
Adequate antenatal care	0.21	0.09	0.49	<0.001

## Discussion

Utilizing timely and appropriate evidence-based strategies during ANC can enhance the well-being of both the mother and the fetus [17]. In contrast, a lack of sufficient ANC and delivery services has been associated with maternal complications and adverse perinatal outcomes [18].

Globally, 62% of pregnant women received adequate ANC services recommended by the WHO in 2017 [19]. However, in Bangladesh during 2017-18, it was only 47% [13]. Over 87% of pregnant women in the present study received adequate antenatal care. This study was conducted at an urban medical college hospital where the majority of patients belonged to affluent and well-educated families. The proportion of people receiving ANC care has nearly doubled compared to the national average, maybe due to the aforesaid factor. The greater percentage (84.5%) of cesarean sections can also be elucidated using the same rationale.

Preterm birth stands out as the most prevalent adverse perinatal outcome noted in this present study. The agreement among numerous research highlights the strength of the evidence connecting inadequate antenatal care to a higher likelihood of premature births [20-22]. There is ample evidence of low-birth-weight newborns and fetal and neonatal deaths among those who did not attend ANC [23,24]. Preterm labor and IUGR may cause low birth weight [25,26]. A significant number of babies born preterm, with low birth weight, or who suffer from IUGR need NICU admission [27,28]. Furthermore, pregnant women with inadequate ANC attendance had a higher likelihood of their infants being admitted to the NICU. Inadequate ANC also has been identified as a contributing factor to perinatal mortality [9]. Lack of ANC education may have prevented early identification and prevention of adverse outcomes in such mothers [29]. Wondemagegn et al. found that ensuring proper ANC for mothers can lead to a significant 34% reduction in the risk of neonatal death [30].

In the current study, a noteworthy significant association emerged, indicating that consistent receipt of ANC had a higher prevalence of having favorable perinatal outcomes. Specifically, women who adhered to adequate ANC demonstrated a higher incidence of normal perinatal outcomes, accounting for 68%, in contrast to those with inadequate ANC, where only 31% experienced normal perinatal outcomes. The data revealed that receiving adequate ANC served as a protective factor against adverse perinatal outcomes. Pregnant women who had adequate ANC were 79% less likely to experience adverse perinatal outcomes than those who received inadequate ANC. This underscores the importance of regular monitoring and healthcare interventions during pregnancy for achieving better perinatal outcomes.

Limitations

This study predominantly relies on observational data, making it challenging to establish a direct causal relationship. It was a hospital-based study, so it might not represent the community scenario.

## Conclusions

Notable adverse perinatal outcomes included preterm birth, admission to the NICU, and perinatal death. While the majority of pregnant women received sufficient ANC, it is worth noting that inadequate ANC was attributed to a notably higher percentage of adverse perinatal outcomes. The high proportion of adverse outcomes can be mitigated by ensuring adequate ANC. Educating mothers and other family members on the value of getting adequate ANC and pointing out that this economic strategy may be the key to improving perinatal outcomes and accomplishing the SDGs.
